# Microalgal-based feed: promising alternative feedstocks for livestock and poultry production

**DOI:** 10.1186/s40104-021-00593-z

**Published:** 2021-06-17

**Authors:** Imen Saadaoui, Rihab Rasheed, Ana Aguilar, Maroua Cherif, Hareb Al Jabri, Sami Sayadi, Schonna R. Manning

**Affiliations:** 1 Center for Sustainable Development, College of Arts and Sciences, Qatar University, P.O.Box.2713 Doha, Qatar; 2 Department of Molecular Biosciences, UTEX Culture Collection of Algae, University of Texas at Austin, Austin, TX 78712 USA

**Keywords:** Functional feed, High-value metabolite, Livestock production, Microalgae, Poultry product

## Abstract

There is an immediate need to identify alternative sources of high-nutrient feedstocks for domestic livestock production and poultry, not only to support growing food demands but also to produce microalgae-source functional foods with multiple health benefits. Various species of microalgae and cyanobacteria are used to supplement existing feedstocks. In this review, microalgae have been defined as a potential feedstock for domestic animals due to their abundance of proteins, carbohydrates, lipids, minerals, vitamins, and other high-value products. Additionally, the positive physiological effects on products of animals fed with microalgal biomass have been compiled and recommendations are listed to enhance the assimilation of biomolecules in ruminant and nonruminant animals, which possess differing digestive systems. Furthermore, the role of microalgae as prebiotics is also discussed. With regards to large scale cultivation of microalgae for use as feed, many economic trade-offs must be considered such as the selection of strains with desired nutritional properties, cultivation systems, and steps for downstream processing. These factors are highlighted with further investigations needed to reduce the overall costs of cultivation. Finally, this review outlines the pros and cons of utilizing microalgae as a supplementary feedstock for poultry and cattle, existing cultivation strategies, and the economics of large-scale microalgal production.

## Introduction

Production of animal feed can be an expensive process; thus, alternative economical high-quality ingredients are desired to supplement conventional feedstocks to meet the growing demands. Traditionally, microalgae have been used as a sustainable resource for domestic livestock, poultry and aquaculture production due to their diverse nutritional profiles, i.e., carbohydrates, essential fatty acids and amino acids, carotenoids, and vitamins [1, 2] Research has shown that blending a small portion of traditional feed with microalgae, e.g., *Chlorella*, *Scenedesmus*, and *Arthrospira*, can positively affect the growth, health, overall animal physiology and product quality and quantity [3]. Moreover, it was stated that microalgae feed-supplement (i) presents cholesterol-lowering effect in animals, (ii) improves immune response [4], (iii) enhances milk quality and production yield in cows [5], (iv) promotes animal growth and improves meat and egg quality [6], (v) offers resistance to disease through antiviral and antibacterial action [7], (vi) improves gut function [8], (vii) enriches the colonization of probiotics [8, 9] and increases feed conversion [10]. Furthermore, it was recently proved that algae-based feed increases reproductive performance and helps in weight control [11].

The use of microalgae as feed supplement is currently being practiced in many Asian countries, including Japan, the Philippines, China, and Korea [12]. Authors reported that currently, there are several microalgae cultivation companies active in China for feed production. Additionally, such initiatives are also under way in Japan, Taiwan, Thailand and India, which are at present in their research and development phase and are increasing in size and number. The use of microalgal feedstocks has also spread to the United States and the United Kingdom [8, 13] In fact, around 30% of the world’s microalgal biomass production is presently sold for animal feed applications [14, 15]. Thus far, more than 30,000 to 40,000 different strains of microalgae have been isolated and classified [16–18], and it is expected that many more will be discovered for potential use in feed. However, there is a long way to go before the production of microalgae-based feed sustainable and economically feasible.

The increasing interest in using microalgae as feed has led to a rise in the research activities and related publications in this field. However, the focus has been on specific applications such as aquafeed of poultry or animal feed. The current paper presents a broad spectrum of various applications which utilize microalgae as feed in poultry and livestock, highlighting several interesting microalgae metabolites with their nutritional and health benefits. Additionally, several challenges and limitations affecting the efficient use of microalgal biomass for feed purpose are also presented and discussed. Furthermore, this literature review considers the economic feasibility of the microalgal feed production based on its cultivation and product value. Hence, providing a comprehensive study of all the parameters included in the process. The review also presents a case study on Qatar, an emerging economy, that needs local alternatives such as microalgae to achieve self-sufficiency on food products.

## Microalgal compounds

**Fig. 1:**
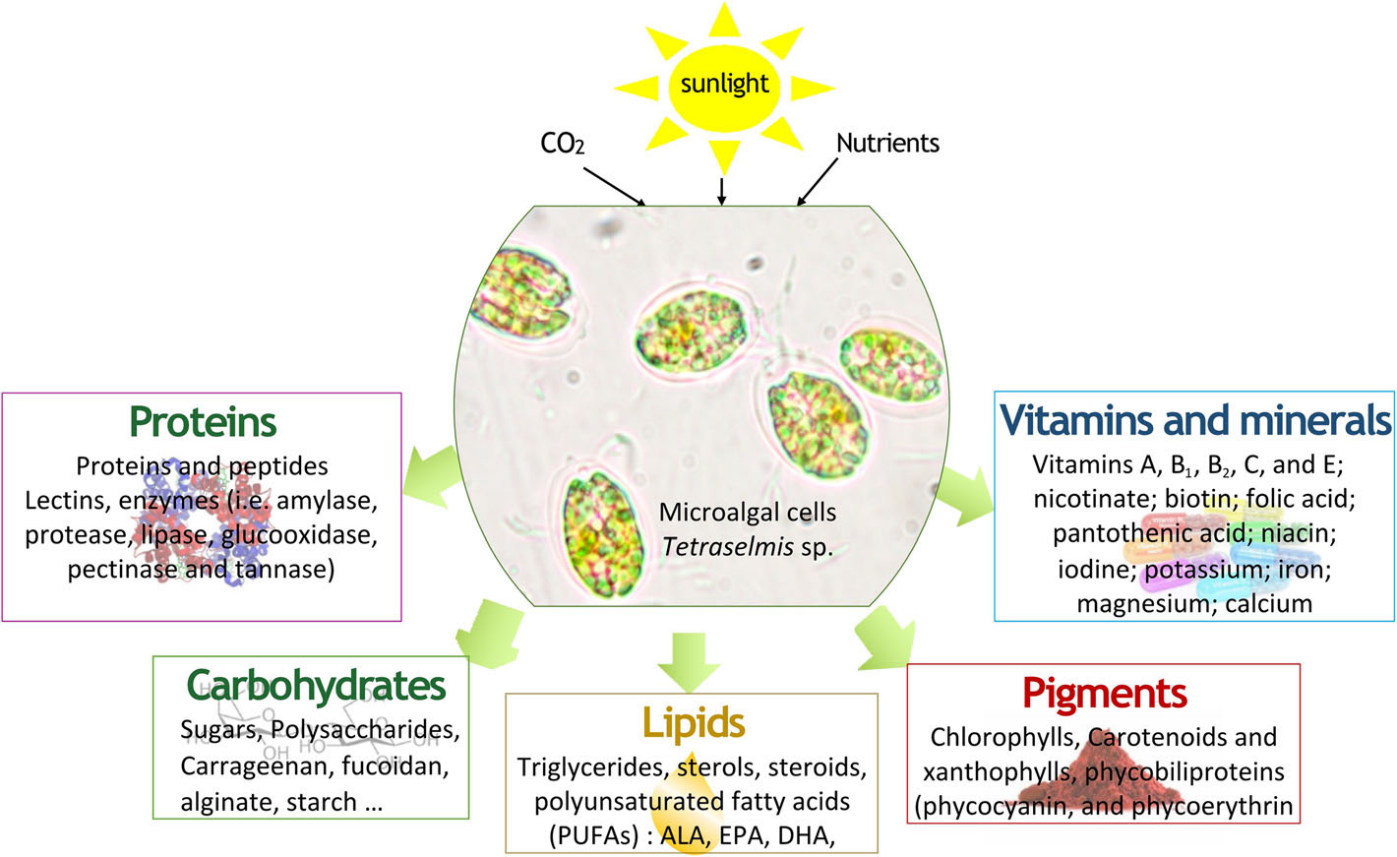
Microalgae metabolites produced during photosynthetic activity

Microalgae are photosynthetic microorganisms that consume atmospheric CO_2_ and light energy to produce a variety of proteins, carbohydrates, and lipids, as well as microelements including, minerals, vitamins, polyphenols, flavonoids, and carotenoids [19, 20] (Fig. 1). The biochemical profiles of the microalgae species commonly used for producing animal feed such as *Arthrospira platensis*, *Dunaliella salina*, *Hematococcus pluvialis*, *Chlorella* sp., *Nannochloropsis granulate* and *Tetraselmis chui*, are presented in the Table 1. microalgae are highly dynamic and vary widely among strains due to differences in growth conditions such as temperature, geographical location, availability of sun light, etc. In addition to known metabolites, microalgae represent a resource of unexploited compounds, that may have unique properties and interesting applications, including but not limited to lipoproteins, sterols and alkaloids [29–31]. These molecules possess several health benefits such as boosting the immune system, which will eventually reduce the use of antibiotics for livestock poultry farming and aquaculture [10, 32, 33].

**Table 1:** Notional properties of the microalgae species commonly used for feed

Microalgae species	***A. platensis,*** % of DW	***D. salina,*** % of DW	***H. pluvialis,*** % of DW	***Chlorella,*** % of DW	***Nannochloropsis granulata,*** % of DW	***Tetraselmis chui,*** % of DW
Crude protein, %	51.4–67	10–30	29–45	20–60	26–33.5	46.5
Crude fat, %	1.8–7.3	10–20	20–25	13.3–20.9	15.3–23.6	12.3
Crude Carbohydrates, %	12.5	250–80	15–17	18.1–27.5	32–36.2	25
Fibre (Total Dietary Fiber), %	1.0	9–11	34–58	16–35	17.67	–
% Amino acids						
Essential Amino acids						
Threonine	2.9–4.9	7.3	5.47	2.5	5.4	4.0
Valine	4.2–4.6	6.3	2.45	3.0	7.1	4.8
Methionine	1.2–1.6	2.1	0.65	1.2	3.5	2.4
Isoleucine	4.2–4.4	5.7	4.32	2.0	5.6	3.4
Leucine	5.5–8.0	7.3	3.64	4.7	11	7.3
Phenylalanine	3.0–5.8	11.5	1.4	2.7	6.2	4.7
Histidine	1.5–2.7	2.6	0.31	2.2	2.3	1.6
Lysine	2.9–3.0	2.1	2.68	4.0	8.5	5.6
Arginine	4.0–4.9	2.6	10.26	3.1	7.4	9.4
Tryptophane	0.1–2.5	2.6	–	1.0	2.8	2.3
Non-Essential amino acids						
Asppartic acid	2.4–9.2	18.2	5.01	4.7	11.4	14.1
Glutamic acid	5.7–10.7	10.4	10.41	5.8	5.6	4.2
Serine	2.8–4.3	0.5	3.43	1.0	14.1	12
Proline	2.0–4.0	1.0	1.24	2.5	11.2	3.6
Glycine	1.8–5.2	9.4	6.61	3.4	7.5	6.5
Alanine	5.4–6.5	7.3	5.6	4.6	7.1	6
Cysteine	0.4–0.5	2.1	0.25	0.7	1.6	2.8
Tyrosine	3.2–3.3	1.0	2.22	2.4	4.2	3
Fatty Acids, g/kg						
ALA	–	21.19	–	–	–	
EPA	< 2.5	–	0.6	< 4.0	34	8
DHA	< 3.0	–	12.8	< 26.0	–	
Elemental Composition						
Minerals, %						
Calcium	1.3–14.0	2.10	0.25	0.1–5.9	0.09	2.99
Phosphorous	1.2–9.6	1.58	1.31	9.6–17.6	0.73	1.46
Potassium	6.4–16.6	4.32	0.97	0.5–21.5		
Magnesium	2.0–3.2	1.37	0.22	3.6–8.0	0.26	0.43
Trace elements, mg/kg						
Manganese	0.02–0.04	–	111.9	0.02–0.10	453	191
Natrium	4.5–10.5	35.4	58.7	13.5	–	–
Iron	0.5–1.8	4.5	822	0.4–5.5	<LD	0.5
Zinc	0.02–0.04	–	232	0.5	0.5	–
Selenium	–	–	–	–	0.5	0.5
Copper	–			–	17.8	102
Carotenoids, g/kg	0330–50	30–100	5–50	8–80	10–60	
Vitamin, mg/kg						
Vit B_9_	0.9			269	–	–
Vit B_1_	5–50			15–24	70	1.09
Vit B_6_	4–50			10–17	3.6	5.8
Vit E	50–190	153.2		200	0.29	1.57
Vit B_12_	–	42–49		–	1.7	–
Ash, %	5.8–9.4	48	4–29	6.2–7.3	8	16.2
Reference	[6, 21, 22]	[23–25]	[23, 26–28]	[6, 23]	[19, 23]	[19]

### Protein

Finding multiple and novel alternative sources of proteins can be of great importance for food security purposes [34]. Different sources of proteins have been consumed in the last decade as food and feed. However, the overuse of such sources has led to the increasing awareness about finding novel alternatives to overcome the protein shortage risk [14]. Microalgae can be considered as a very promising alternative. Some strains of microalgae produce high amounts of protein and could be a potential renewable resource for feed supplements [31, 32], e.g., Cyanobacterium *Arthrospira* can contain up to 70% protein. Microalgae present very similar essential amino acid profiling to that of superior vegetable proteins such as Soya bean [19, 35, 36]. According to FAO/WHO recommendation, Microalgae such as *Chlorella* and *Arthrospira* (Spirulina) are considered as sustainable source of proteins suitable for human consumption due to the presence of essential amino acids similar to those acquired from conventional protein sources such as soybean and egg (FAO/WHO, 1991). Feed stocks containing higher concentrations of methionine and lysine have been found to increase chicken breast and thigh muscles and improve the quality and quantity of meat. Microalgae also produce bioactive peptides with antioxidative, antihypertensive, anticoagulative, antitumor and immune-simulative properties [11, 36]. The most widely used microalgae for protein-rich feed supplements include species of *Chlorella*, *Arthrospira*, *Dunaliella*, *Tetraselmis*, *Phaeodactylum*, *Skeletonema*, and *Scenedesmus* [37, 38].

### Carbohydrates

Carbohydrates are a significant component of microalgae due to their nutritional and pharmaceutical value. Indeed, beta-1-3-glucan, a type of soluble fiber, most importantly found in *Chlorella* sp., is an antioxidant that helps in lowering cholesterol levels in blood [37, 39]. Moreover, A promising microalgae for the commercial production of carbohydrates is the unicellular red alga *Porphyridium cruentum*, which produces a sulfated galactan exopolysaccharide and can replace carrageenan in the meat and dairy industry [40]. Xylose, mannose, glucose, galactose and rhamnose are the most common species dependent monosaccharides which can be obtained through the production of the microalgal polysaccharides [41]. Among these sugars, glucose is highly detected in several green microalgae species with 47–85% of the total carbohydrates [41]. However, mannose is detected in higher concentrations in diatoms reaching 45.9% per total carbohydrates in the case of *Phaeodactylum tnicornutum*.

### Lipids

Several microalgae species have been considered as excellent source of dietary lipids. Depending upon the strain and culturing conditions, microalgae can produce up to 50% lipids (w/w) on a dry weight (DW) basis, and occasionally even more [17, 42]. The long-chain fatty acid profile of some microalgae can be enriched with polyunsaturated fatty acids such as eicosapentaenoic acid (EPA), alpha-linolenic acid (ALA), arachidonic acid (AA), docosahexaenoic acid (DHA), and linoleic acid (LA) [43]. These omega fatty acids are essential and cannot be synthesized by humans and animals, thus they must be ingested and absorbed. Furthermore, DHA and EPA are known with a range of biological functionalities such as antioxidant and anti-inflammatory activities, improving mental health, reducing the risk of cardiac diseases like arrhythmia, stroke, rheumatoid arthritis, and high blood pressure [20, 44]. Recent studies proved that supplementing infertile men with omega-3 fatty acids resulted in a significant improvement in sperm motility and concentration of DHA in seminal plasma [45]. Several microalgae species are known to be good source of these essential fatty acids such as *Isochrysis*, *Nannochloropsis*, *Tetraselmis* and *Arthrospira* [46–48].

### Carotenoids

Carotenoids provide nutritional, therapeutic, and antioxidant properties [49]. Carotenoids are typically used as food-coloring agents and there are around 200 carotenoids which can be sourced from microalgae. Among them, β-carotenes and astaxanthin represent the most commercially produced carotenoids [50–52]. Other carotenoids such as lutein, zeaxanthin and lycopene are lesser known despite their interesting nutritional value (Table 2). In poultry feeding trials, microalgal feedstocks enriched with β-carotene led to a dark-yellow color of the yolk [60]. While microalgal carotenoids are more expensive than synthetic forms, natural sources provide more isomers [60]. *Dunaliella salina* is commonly used for β-carotene production, since they can accumulate up to 14% of their weight as β-carotene, under extreme conditions such as hypersaline, low nitrogen, and high levels of solar irradiation [13, 61, 62]. Similarly, strains of *Haematococcus pluvialis* produce up to 4–5% astaxanthin, including free, mono-, and di-ester forms, under stressed conditions [23, 26, 63].

**Table 2:** Common carotenoids found in microalgae

Carotenoids	Microalgal Species	References
β-carotene	*Dunaliella salina* and *D.bardawil*	[25]
Astaxanthin	*Haematococcus pluvialis, Chlorella zofingiensis*	[26]
Lutein	*Scenedesmus almeriensis*	[25]
Canthaxanthin	*Chlorella zofingiensis*	[52]
Lycopene	*Chlorella marina*	[53]
Fucoxanthin	*Phaeodactylum tricornutum*	[54]
Zeaxanthin	*Microcystis aeruginosa*	[55]
Alloxanthin	*Cryptomonar ovata*	[56]
Antheraxanthin	*Chrysophaera magna*	[57]
Violoxanthin neoxanthin	*Chlorella vulgaris, Scenedesmus quadricauda, Neochloris oleoabundans, C. protothecoides*	[58]
Peridinin	*Dinophyta*	[59]

### Vitamins and minerals

Microalgal biomass represents a valuable resource for many essential vitamins and could be used to supplement feed stocks [64]. These include vitamin A and other retinoids, B vitamins like thiamine (B_1_), niacin (B_2_), nicotinate (B_3_), pantothenic acid (B_5_), pyridoxal phosphate (B_6_), biotin (B_7_), folic acid (B_9_) and cobalamin (B_12_), vitamin C (ascorbic acid), vitamin E (tocopherols), and a variety of trace metals and minerals (e.g. sodium, potassium, calcium, magnesium, iron, and zinc) [6, 8, 65] Microalgae also synthesize vitamins and accumulate minerals in their natural forms so that they can be easily assimilated, when compared to synthetic forms [66]. The synthesis of vitamins depends on the strain, light intensity, nutrients available in the media, and stage of the growth cycle, among other factors. Strains of *Tetraselmis* sp. have shown high amounts of thiamine, pyridoxine, nicotinic acid, and pantothenic acid, whereas strains of *Dunaliella* contained elevated amounts of β-carotene, riboflavin, and cobalamin. Some isolates of *Chlorella* were found to contain abundant levels of tocopherol and biotin [64] (Table 1).

## Nutritional potential of microalgae-based feed

### Microalgae for animal feed

**Fig. 2:**
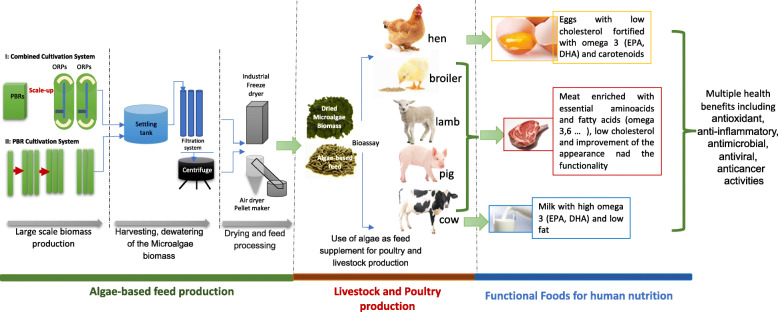
Technology process lineup for producing animal-sources functional foods via using algae-based feed

Microalgae can be the next alternate source for animal and aquatic feed production in an environmentally sustainable and nutritionally beneficial manner [15]. These microorganisms can be produced in large scale using photobioreactors and open ponds then harvested and processed to produce functional feed supplements for different animals such as poultry, pigs, sheep and mink [67, 68]. The Fig. 2 represents the technology process lineup for producing high quality algae-based feed for animal-sourced functional foods. Altering the cultivation conditions can make the feed beneficial, since it can be enriched with highly valuable metabolites such as omega fatty acids, carotenoids and essential amino acids [11, 50, 69, 70]. Consequently, the enriched microalgae can be used as feed supplement to improve the quality of their meat, eggs and milk products which will provide multiple health benefits such as anticancer, antioxidant and antiviral effects to humans when consumed [20, 71, 72] (Table 3).

**Table 3:** Bioavailable energy values of algae-based feed for livestock and poultry

Algae species used for animal feed	Animal	ME (metabolizable energy)	Net energy (NE)	Ileal Digestibility Coefficient for essential AA	Reference
*Schizochytrium* spp.	Cow	3.48 Mcal	–	–	[73]
*Arthrospira platensis* (Spirulina)	Broiler Chicken	13.48 MJ/kg DM	18.4 MJ/kg	0.80 ± 0.04 MJ/kg DM	[21]
*Arthrospira platensis*	Poultry	3.48 Mcal	–	–	[22]

### Microalgae feed supplement for eggs production

Literature related to the manipulation of polyunsaturated fatty acids (PUFAs) in the diet is extensive, which has led to the production of customized egg yolks that are rich in PUFAs to meet desirable nutritional characteristics [74, 75]. Microalgae are an alternative feedstocks for essential omega-3 fatty acids (ω-3 FAs) to improve the nutritional value of eggs for human consumption [76]. Many ω-3 FAs have potent anti-inflammatory properties that are essential for brain development and maintenance, as well as for the prevention of cardiovascular disease. Laying hens fed with diets supplemented with *Nannochloropsis gaditana*, containing long-chain ω-3 FAs, such as eicosapentaenoic acid (EPA), docosapentaenoic acid (DPA), and docosahexaenoic acid (DHA), resulted in the accumulation of these ω-3 FAs in the egg yolk [77]. The abundance of ω-3 FAs in eggs varies with chicken age and breed, and microalgae digestibility [75, 78].

Eggs from hens that were fed with algal-blended feedstocks enriched with ω-3 FAs contained more beneficial fatty acids when compared to eggs produced from conventional feed [79]. These long-chain fatty acids are almost exclusively located in the phospholipids of the yolk. In feed trials, very small quantities of microalgal biomass added to feedstocks yielded significant changes in the ω-3 FA content of eggs. Additionally, Herber and Van Elswyk [80] and Moran et al., [81] demonstrated that the efficiency of DHA assimilation from microalgae to eggs was 42.6% in hens when they were provided with a 2.4% algal-blended feed. Compared to the control eggs, the DHA content increased 6-fold in eggs from hens fed with algae–based feed [76]. Feedstocks high in EPA with trace levels of DHA (as is found with *Nannochloropsis oculata*) tend to produce eggs that are low in EPA and high in DHA, indicating fatty-acid chain elongation of EPA to DHA or the preferential bio-assimilation of DHA over EPA. It is noteworthy that, chickens supplemented with algal feedstocks such *Porphyridium* sp. consumed 10% less feed than the control groups, and the egg yolks had reduced cholesterol levels and lower ratios of ω-6:ω-3 FAs [82]. Omega-rich feedstocks comprised of 2.5% dried-fermented *Schizochytrium* sp. blended with flax seeds yielded 150 mg of ω-3 FAs/egg [83]. While seedstocks containing substantially high levels of ω-3 FAs have no adverse effects on the performance or health of the bird, it should be noted that omega-rich feedstocks can cause a decrease in the tocopherols necessary for proper egg yolk formation and oxidative stability [84]. It is reported that micro algal inclusion as low as 1.5% to 10% is beneficial for broilers, beyond which the growth and quality of eggs will be negatively affected [79].

Fredriksson et al. [79] tested the addition of 20% *Nannochloropsis oculata* in hen feedstocks. In their examinations, the lutein and zeaxanthin content of the eggs was approximately 1.3 mg/egg after 4 weeks of feeding hens a diet supplemented with microalgae. Egg yolks with higher concentrations of carotenoids often appeared dark orange to red [85]. Both synthetic carotenoids (e.g., Carophylls) and natural carotenoids can significantly increase the egg weight and improve feed conversion. However, naturally-occurring lutein in *Chlorella*-supplemented feedstocks was found to be incorporated more efficiently and significantly increased the oxidative stability of yolk lipids [86]. Furthermore, diets rich in carotenoids improved eggshell thickness and other desirable physical properties.

### Microalgae for meat quality

Omega-3 fatty acids are considered essential because humans and livestock lack the ability to synthesize them, and they must be obtained through diet. The health benefits of PUFAs, including ALA, EPA, and DHA have been well documented [87]. Consequently, it has been suggested that consuming foods with significant amounts of ω-3 PUFAs can have significant health benefits and these compounds have high commercial potential [88].

A high percentage of PUFAs are bio-hydrogenated in the rumen if the feedstocks are unprotected (i.e., uncoated). The diet of ruminant animals is based on cereals or forage known to be rich in linoleic acid (LA, C18:2 n-6) and ALA (C18:3 n-3) [89], although very low concentrations of these PUFAs were observed in the ruminant meat [90]. In fact, about 70–95% of LA and 85–100% of ALA are bio-hydrogenated before leaving the rumen [90, 91]. Several strategies have been adopted to enrich meat with PUFAs, especially EPA and DHA, such as supplementing conventional animal feed with fish, marine microalgae, and algae-like microorganisms. Such bioassays proved that the algal-based feed supplement was the best at enhancing EPA and DHA levels in the animal meat. On the other hand, Elmore et al. [92] proved that using linseed and fish oils as feed supplements improved the meat quality in Suffolk and Soya lambs by doubling the amount of ALA and increased EPA and DHA at 2- and 4-fold, respectively.

It has been shown that mixing one or more PUFA-rich algal species such as *Arthrospira maxima* and *Arthrospira platensis*, or *Chlorella* sp. into the diet of pigs produced meat with a well-balanced lipid profile [36, 93]. More recently, it was proved that using microalgae, such as *Schizochytrium* sp., as feed stock, with an inclusion percentages of 5% and 7%, used for growing pigs enhanced the omega 3 fatty acid content in their meat with inducing changes in skeletal muscle, phenotypic appearance and functionality [94]. In a different study, it was shown that including microalgae in the diets of weanling pigs by up to 33%, which is so far, the highest amount recorded, did not affect them negatively. However no weight gain was also reported [95].

In the case of poultry meat, it was previously described by Toyomizu et al. [96] that supplementing of the conventional poultry feed with *Arthrospira* (4% or 8%), did not show any effect on the growth performance of the broilers but it led to the yellowness of muscles, skin, fat and liver, which adds to commercial value of the meat in the market. However, supplementing poultry feed with fresh liquid algae (1%) improves the body weight gain, immune characteristics, and production of *Lactobacillus* bacteria in the intestinal microflora of broiler chickens [97]. Finally, defatted biomass of *Chlorella* and *Arthrospira* obtained from biofuel production showed positive effect on poultry meat quality [98].

### Microalgae for milk production and quality

Increasing research exists on the use of microalgae as a dietary supplement for the accumulation of beneficial fatty acids in milk [5, 73, 99–101]. However, the effects of microalgal metabolites on lactation and the transfer of nutrients to the milk largely depends on the animal’s digestive system (ruminant vs. non-ruminant) as well as the biosynthetic capabilities of the animal [5]. In case of ruminants, the nature of enzymes in the small intestine and the rumen microorganisms, have an impact on the digestion and absorption of fatty acids from the intestine. The intestinal and microbial enzymes break down the unsaturated fatty acids into short chain saturated fatty acids for absorption, resulting in the modification of the nature of molecules that will be incorporated into the animal tissue. While in non-ruminant animals, dietary fatty acids are unchanged and can be absorbed by the small intestine and incorporated directly into tissues.

It has been demonstrated that milk quality is strictly influenced by the type and abundance of fatty acids consumed by cattle, thus bio-hydrogenation in the rumen must be prevented [102]. It has recently been proved that microalgae are likely comparable protein feed to soya bean meal in dairy cattle nutrition [99]. Thus, it is recommended to use coated microalgae biomass to protect the nutritional properties of the feedstock in ruminant animals, allowing more ω-3 FAs to be absorbed by the small intestine which will then be transferred to the mammary glands [103]. Additionally, blended algal feedstocks have shown to increase the abundance of LA, DHA, and vaccenic acids in the milk fat [99, 104, 105].

Microalgae supplementation has shown to increase the DHA content up to 4 times in milk [106]. The most commonly used microalgae strains for improving the quality of milk in terms of useful fatty acids are *Schizochytrium* and *Nannochloropsis* [101, 104]. Additionally, *Nannochloropsis* sp. has also showed a higher content of EPA along with other PUFAs [107], in comparison with strains such as *Arthrospira platensis* (cyanobacterium) and *C. vulgaris* [42], showing EPA contents high as fish oil [99, 108]. Such findings state that microalgae belonging to different genera differ in their biochemical profiles and will have different effects on animals when used as feedstocks.

Despite the increased quantities of LA, EPA, and DHA in milk enriched with ω-3 FAs, the oxidative stability of the milk remains unchanged [106, 109]. In addition, feeding ω-3 FAs during lactation was found to reduce prostaglandin secretion, which can improve fertility and embryo survival [100]. Additionally, incorporating microalgae at 5–10% inclusion rates while feeding livestock, enhances the mineral content such as for Iron, Iodine, potassium and zinc found in the milk and meat of the animals [65]. Finally, Glover et al. [106] demonstrated the rumen-protected microalgae reduced total milk solids (12.57% vs. 13.19% ± 0.17%; *P* = 0.02) and the milk-fat content (3.99% vs. 4.70% ± 0.17%; *P* = 0.007). The urea content in milk was also lower for cows fed protected microalgae (2.98 mg/dL vs. 3.22 ± 1.27 mg/dL; *P =* 0.01).

### Microalgae as prebiotics

Prebiotics prevent pathogen invasion in the body via boosting the immune system, resulting in enhancing the animal’s health [110]. The most promising feed ingredient conferring prebiotic properties are polysaccharides or their derivatives, which include the dietary fibers [111]. Currently, large number of microalgae presenting prebiotic effect are being used in feed industry. *Chlorella* sp. produced an acidic polysaccharide containing rhamnose (52%) along with arabinose and galactose. This complex is known to have immune-stimulating properties by inhibiting the proliferation of harmful pathogens [112]. Similarly, the cell walls of *Tetraselmis* sp. also consists of acidic polysaccharides (82% DW) favoring the gut microbiota. Another interesting microalga is *Dunaliella salina*, which reportedly produces extracellular polysaccharides known to have immunostimulatant, antiviral and antitumor properties [24]. Therefore, it is evident that microalgae not only improve the health and performance of animals by direct supply of nutrients, as discussed in the previous sections, but also benefit them indirectly, by promoting the intestinal microbiota that enhances the animal’s health.

## Limitations of using microalgal feedstocks

The current challenges in food industry are to find alternative sources of nutrients and additives that can add value to the agricultural products and protect them from oxidation. Researchers have successfully incorporated PUFAs into numerous foods [113, 114]. However, Shingfield et al. [91] proved that the accumulation of PUFAs can affect the flavor of the meat and milk in addition to the increased risk of oxidation. Lee et al. [114] also demonstrated that increasing PUFAs in meat affects its flavor and routine meat processing procedures (e.g., grinding and cooking) by intensifying lipid oxidation.

Moreover, milk and dairy products enriched with PUFAs are more susceptible to oxidation [65, 115] which can ultimately have an effect on the milk quality [115–117]. However, this can be slowed down by using antioxidants, including tocopherols and carotenoids, and thus preserve the quality of milk. Several investigations have demonstrated that an antioxidant combination, such as a radical quencher, chelator (e.g., sodium citrate), or reductant (e.g., sodium erythorbate) can be added as feed supplement to fortify the meat, minimizing lipid oxidation [114], enhance PUFA absorption, facilitate easy incorporation into tissues [88], while preserving the color of the meat, and maintain the concentration of PUFAs during storage [114].

The use of microalgae in feed provides the necessary nutrients such as PUFAs in addition to antioxidants such as carotenoids, for enriching and preserving food. However, selection of the microalgae species to produce the feed likely affects the animal performance as the species can significantly differ in terms of metabolic composition, protein degradability and cell wall composition [21]. Digestibility is affected by (i) the high fiber content in the cell wall [118], (ii) the high polysaccharides content reaching up to 64% of the DW with specific species and under specific cultivation conditions, and (iii) high phenolic compounds that can react with amino acids to form insoluble compounds [1] (Table 2). Hence, several methods have been recently optimized to treat the cell membrane in order to release the valuable intracellular contents [21]. Finally, it is also essential to identify the range of microalgae inclusion in the diet as performed by Evans et al. [119], where *Arthrospira* was tested in different proportions (6–21%), positive impact on poultry meat was observed only with feed amended up to 16% microalgae. This could be explained by the low digestibility of high microalgae amounts in the diet [68]. More recently, 12 microalgae have been characterized for biochemical composition and “*in vitro*” digestibility. Results proved that *Arthrospira* and *Chlorella sorokiniana* with a high protein content between 50% and 65% exhibited the highest digestibility [22]. However, *Tetraselmis* strains rich in fibers and lipids showed the lowest digestibility. This was explained by the presence of robust cell walls or of exopolysaccharides that might have limited the action of digestive enzymes [22]. Additionally, Moheimani et al. [120] proved similar digestibility various combinations including ground, ground plus bead-milled, and ground plus bead-milled plus defatted microalgal biomass via “*in vitro*” assays. However, fermented *Chlorella* improve nutrient digestibility, fecal microbial shedding and the growth performance of 6 weeks old pigs when compared with the conventional diet. More recent research proved that poultry diet containing up to 16% of *Arthrospira* biomass lead to high digestibility of cysteine and lysine [119] (Table 3).

## Challenges and achievements in producing microalgal feedstocks

### Cultivation and production of biomass

The technologies needed for cultivation, harvesting, and processing/extraction are considered as leading factors contributing to the high costs of producing microalgal biomass [121]. The most common growth systems are open raceway ponds, tubular photobioreactors (PBRs), and flat-panel PBRs. The selection of a large-scale commercial culture system is dependent on several parameters such as cell biology, land availability, operating, production, harvesting and maintenance costs, energy and nutrient requirements, water availability and climate conditions [122]. Raceway ponds are typically shallow, ring-channel systems where cultures are mixed with a paddle wheel set at a fixed velocity. It was stated previously that open-pond systems generate low-density biomass, ranging from 0.3–0.5 g/L DW [123, 124]. While this technology uses much less energy for mixing cultures than the other reactor designs, higher costs are incurred for harvesting due to the low culture densities. Moreover, the cultures grown in this system are significantly affected due to external environmental factors such as temperature, pH, light, salinity and pollution, which also explains the low productivity [125]. Sugar-stimulated CO_2_ sequestration by the green microalga *Chlorella vulgaris* [125]. Open ponds are used extensively in industrial microalgal production where huge volumes of culture (around 40,000 L in 200 m^2^ illuminated area) are maintained [126]. Such cultivation system generated an annual production of *Arthrospira* and *Dunaliella* biomass of 3,000 tons and 1,200 tons, respectively [37].

In tubular PBRs, algal cultures are circulated in semi-closed transparent glass or PVC piping without a centrifugal pump as this damage most algae cells [127]. Typically, biomass densities of greater than 1.7 g/L DW can be obtained using tubular PBRs [124]. Tubular PBRs are commonly used for growing more controlled cultures of microalgae species with high nutraceutical potential such as *Haematococcus*, *Chlorella*, and *Nannochloropsis* [128]. A flat-panel PBR is a transparent, rectangular cuboid vessel in which mixing is carried out by sparging air directly into the reactor. This configuration resulted in biomass productivity of 15 g/m/d of *Tetraselmis suecica* corresponding to an annual yield of 36 tons of dry biomass per hectar [129]. Flat-panel PBRs are ideal for the production of algal strains that accumulate lipids under nutrient limitation stress with the shorter light path found in panel PBRs.

### Economic feasibility of microalgae production and market values

**Fig. 3:**
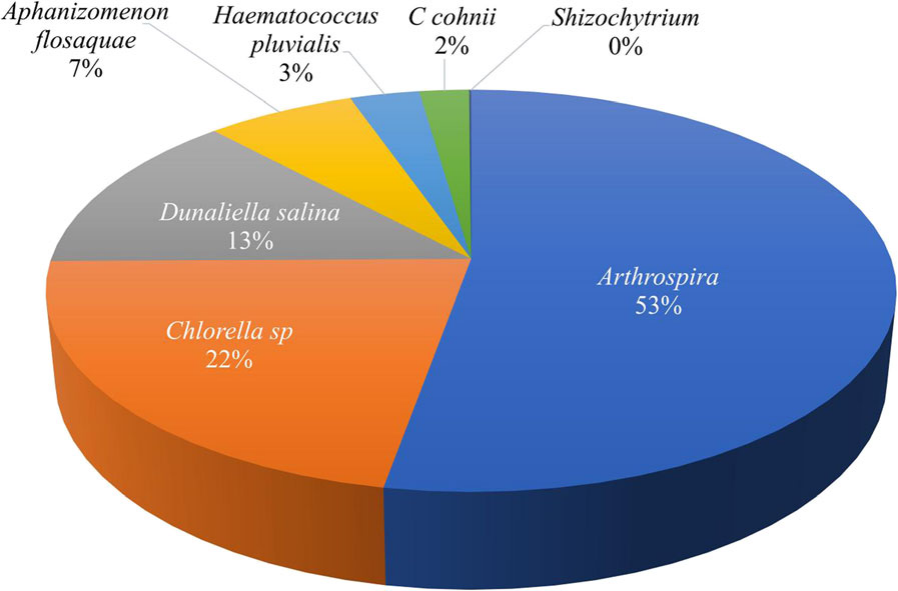
Annual commercial production of the major genera of microalgae worldwide

Microalgal biomass has been proposed as a renewable resource for the generation of energy and other commodities due to their high rates of productivity. They can be grown using low quality water and do not require arable land [62, 91, 130]. Even so, production of microalgal biomass is more expensive when compared to other feedstocks, e.g., wheat sells for $ 0.035 /kg [131]. Improvements in microalgal processing methods together with optimizing the cultivation and harvesting systems are needed to develop technical solutions that will improve the feasibility of cultivating microalgae for biomass and valuable compounds in a profitable means at commercial scale. Currently, the microalgal biomass market produces nearly 5 kt per year and the production costs are around $ 25,000 per ton of biomass from mainly five taxa [132] (Fig. 3).

**Fig. 4:**
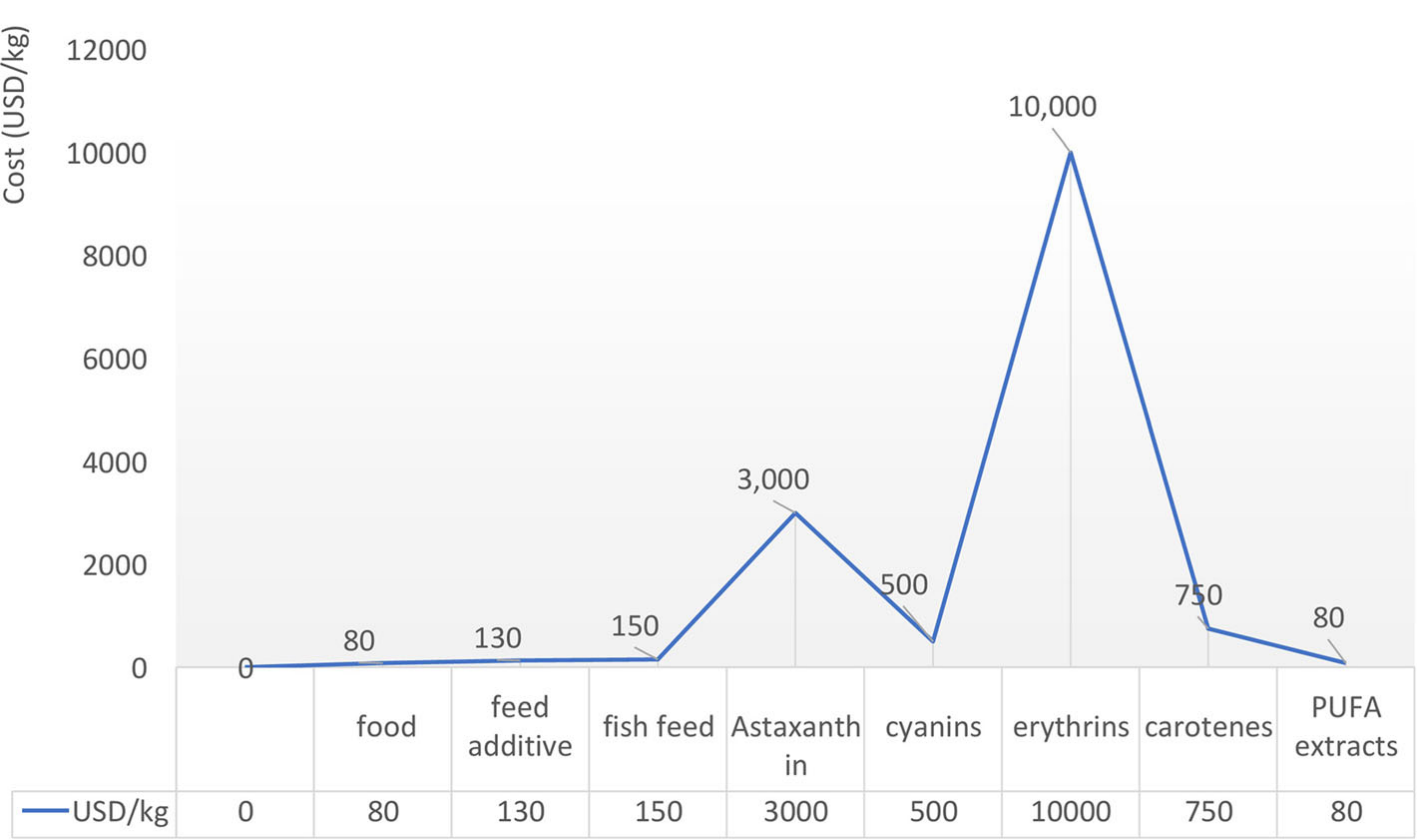
High-value products from microalgae (in USD/kg) for various commercial uses

Biomass recovery cost represents 20–30% to the total production costs [121, 133]. Harvesting the biomass challenging because of the size of the microalgal cells, which range from 3 to 60 μm in diameter. The microalgal biomass is typically dewatered and concentrated using centrifugation, filtration, or in some cases, gravity sedimentation, and each of the processes have different energy demands. These processes may be preceded by a sedimentation step using caustics or flocculants (e.g., alum, chitinase, magnesium hydroxide) to pre-concentrate the biomass for subsequent dewatering [134]. However, it was proved that the chemical flocculants can alter the quality of the final product and/or affect the biomass processing (lipid extraction) [135]. Unfortunately, there is no harvesting method that works for all types of microalgae, and this step must be determined empirically for each strain, including considerations for the application. The annual production of dry algae biomass was estimated of 19,000 tons, generating a revenue of about $5.7 billion [31]. Although production estimates are available for only a few strains of microalgae that are cultivated at the commercial scale: the annual production of *Arthrospira* dominates the market (3000 tons /year) followed by *Chlorella* (2,000 tons/year) [27], *Dunaliella* (1,200 tons/year), *Aphanizomenon* (500–600 tons/year) and *Haematococcus pluvialis* (300 tons/year). This biomass is processed to generate a number of products that are used for various applications, including nutraceuticals, pharmaceuticals animal feed, aquaculture, human food, coloring substances and antioxidants. The global algae market is projected to reach $ 1.143 billion by 2024 with an annual expansion rate of 7.39%. Purified microalgal products, including ω-FAs, antioxidants, and coloring agents, generate significantly more revenue than the unprocessed, whole biomass [28] (Fig. 4). For example: Microalgae such as *Chlorella* and *Scenedesmus* produce a variety of natural functional ingredients such as lutein [136]. The global market for lutein is expecting to reach $ 357.7 million by 2022. Similarly, the global market value for carotenoids reached up to $ 1.24 billion in 2016 and is expected to reach $ 1.53 billion by 2021 [137].

The potential for microalgae to supply the world market is very high although there are still gaps between current production capabilities and market demands. The high production cost of microalgae [138] makes them currently an uncompetitive feed option, but the situation may change in the near future due to technical development and different policy interventions such as incentives and carbon taxation. As per the prnewswire website (https://www.prnewswire.com/news/reportlinker), global algae products market stood at $ 9.9 billion in 2018 and is projected to grow at a Compound annual growth rate (CAGR) of over 7% during 2019–2024.

### Industrial scale production of microalgae for feed application

Microalgae can be cultivated in different systems, depending upon the application of the biomass. For feed purposes, industrial bioreactors and open ponds are suitable [139]. Several studies have shown that microalgae cultivation for feed production at industrial scale is possible and can be achieved from a sustainability perspective. Biomass can be generated when cultivated on wastewater such as from fish processing industry, as explained by Trivedi et al. [140] where *Chlorella vulgaris* was effectively cultivated in untreated water from the industry without the addition of nutrients. It is, however, essential that the final biomass is free from pathogens, toxins and thus is safe for use as feed. In another scenario, sequestering atmospheric CO_2_ to produce microalgae biomass will benefit the environment in addition to improving the production of microalgae. In both the above cases, micro algal production will lead to a cleaner environment and use sustainable resources for growth. Considering the information available, despite the existing knowledge and facilities, use of microalgal products still faces some drawbacks in terms of technological and economical facets. This is, however, likely to be reversed due to its benefits and growing popularity as feed stock.

## Microalgae-based feed production: a case for Qatar

The nutritional content of microalgae makes it a suitable alternative for feed supplement. However, the future use of microalgae in feed formulations depends on development of cost-effective strategies for large-scale production. This is especially important for growing populations that are reliant on imported food. The food market has been seriously challenged over the last few decades by the population growth in Qatar, a country that is overwhelmingly reliant on imported food, which can represent up 90% of the population’s needs – this is neither economically-feasible nor sustainable. Furthermore, regional food production is limited due to arid climate, availability of arable land, and the scarcity of fresh water. Thus, there is an immediate need to identify alternative sources of food to sustain the population of Qatar and meet growing demands.

A country like Qatar, which is located on a peninsula with abundant sunshine, favorable temperatures for cultivation, and access to plenty of water, may be able to co-locate and utilize local resources, thus bringing down the costs of producing microalgal biomass for nutritional feed supplements. Microalgae are already used in many applications in Qatar, including for biofuels, feed, biofertilizers, waste water management, and CO_2_ sequestration [141–147]. The use of microalgae-based foods has gained interests over the last few years, supporting the possibility of using microalgae for supplementing local poultry and livestock to produce enriched animal products for human consumption. Although, as with any case, a technoeconomic analysis would need to be performed, looking at broad suite of variables, to determine economic feasibility.

## Conclusion

Microalgae have tremendous potential as animal feed due to the presence of essential biomolecules such as amino acids, PUFAs and high-value products such as carotenoids and vitamins, that enhance the nutritional quality of the animal products. Consequently, serving as a sustainable source of nutrition for animals. Although these microorganisms are considered as the most suitable alternative, there are certain limitations in using them. Digestibility and selection of the right inclusion dose is some of the challenges that should be addressed with regards to the animal feed. Additionally, the economic feasibility of producing large amounts of biomass for feed is ambiguous due to high production costs, downstream processing, and storage issues. These issues need to be addressed in order for the microalgal feedstock to be cheaper than the existing agricultural products. Since the demand of microalgal biomolecules is growing in the market, more research should be directed towards the cultivation strategies, for identification of sustainable and economical production of biomass for the usage as animal feed and therapeutics.

## Data Availability

Not applicable.

## Abbreviations

*A. platensis*: *Arthrospira platensis*
Aa: Aminoacids
AA: Arachidonic acid
ALA: Alpha-linolenic acid
*C. cohnii*: *Crypthecodinium cohnii*
*C. vulgaris*: *Chlorella vulgaris*
CAGR: Compound annual growth rate
*D. salina*: *Dunaliella salina*
DHA: Docosahexaenoic acid
DPA: Docosapentaenoic acid
DW: Dry weight
EPA: Eicosapentaenoic acid
FA: Fatty acid
FAO: Food and Agriculture Organization
LA: Linoleic acid
ME: Metabolizable energy
NE: Net energy
Pbr: Photobioreactors
PUFAS: Polyunsaturated fatty acids
PVC: Polyvinyl chloride
Vit: Vitamin
WHO: World Health Organization
ω-3 FAs: Omega 3 fatty acid
